# The development of a 3D colour reproduction system of digital impressions with an intraoral scanner and a 3D printer: a preliminary study

**DOI:** 10.1038/s41598-019-56624-3

**Published:** 2019-12-27

**Authors:** Yushu Liu, Rui Zhang, Hongqiang Ye, Shimin Wang, Kuan-Paul Wang, Yunsong Liu, Yongsheng Zhou

**Affiliations:** 1 Department of Prosthodontics, Peking University School and Hospital of Stomatology, Beijing, 100081 China; 2 Department of Prosthodontics, Stomatology Center, Peking University Shenzhen Hospital, Shenzhen, 518001 Guangdong China; 3 Department of Prosthodontics, Peking University School and Hospital of Stomatology, National Clinical Research Center for Oral Diseases, National Engineering Laboratory for Digital and Material Technology of Stomatology, Beijing Key Laboratory of Digital Stomatology, Beijing, 100081 China

**Keywords:** Aesthetic dentistry, Fixed prosthodontics

## Abstract

This study aimed to develop a three-dimensional (3D) colour reproduction system to improve the aesthetic effects of dental prostheses. The system’s colour accuracy was also evaluated. Based on the concept of colour management, 96 colour patches were selected to develop colour profiles for an intraoral scanner and a 3D printer using polynomial regression. The colour differences Δ${E}_{ab}^{\ast }$ between colour patches reproduced using different colour profiles and the original colour patches were analysed to select the best combinations of colour profiles. The 3D colour reproduction system with the best-performing (i.e. third-order polynomial regression) colour profiles was finally evaluated using tooth and gum shades. The median Δ${E}_{ab}^{\ast }$ was 6.940 ranging from 1.504 to 32.660. In terms of tooth and gum shade, the median Δ${E}_{ab}^{\ast }$ was 6.313, and half of the shade blocks were above the mismatch threshold (Δ${E}_{ab}^{\ast }$ > 6.80). In conclusion, the colour management based on polynomial regression can decrease the colour difference of the 3D colour reproduction system, but not to clinically acceptable levels. Further advances are needed to improve the methods and hardware.

## Introduction

In aesthetic dentistry, colour plays an important role alongside tooth morphology and position in achieving a pleasing and natural-looking prosthetic result. The measuring and transferring of intraoral colour information are fundamental to achieve perfect mimicry of the natural teeth and the surrounding tissue, which is essential for aesthetic restorations. In digital dentistry, these processes are still limited by traditional methods. The digitally acquired colour information is mostly used in visualization of the design process, and not often transferred directly to the final prostheses or digitally fabricated dental models.

There are several ways of measuring colour information. A frequently used example is visual shading using shading guides. This is a subjective process and lacks accuracy in certain situations, as many factors can affect its results such as ambient illumination, the patient’s make-up, and the dentist’s colour perception. Another method is digital shading by a digital shading instrument, which is objective and quicker^1–3^ than conventional visual shading. However, these two methods are lossy to a certain extent as they “compress” the colour information of the tooth surface into a single notation (e.g. A2, 2L1.5 and ND2). Clinicians often need to provide technicians with chromatic maps or clinical photographs containing more details^4^, and achieving naturally coloured prostheses remains challenging. The correct interpretation of the chromatic maps relies on good cooperation between clinicians and technicians. The colours of photographs captured with different devices are usually device-dependent, and the colour of a photograph may be different on different displays. Recent research has reported using neural networks to match natural teeth directly with ceramic restorations, but the results were somewhat unstable^5^. The shortcomings of these methods mean a simple, visualized and device-independent method would be favourable.

Recent developments of digital impression technologies allowed the capture of not only the contour but also the detailed colouring of patients’ teeth. Digital impression systems employing real-colour scanning include shading modules capable of demonstrating acceptable accuracy and repeatability^6–8^. Meanwhile, the development of additive manufacturing has already realized full-colour three-dimensional (3D) printing of objects with colours based on the textures of digital models. This technology has been applied in artistic and industrial design for prototype verification. By combining real-colour scanning and full-colour printing, dental practitioners may transmit colour information using chromatic physical dental models that are simple, visualized and device-independent.

However, as scanning and printing systems each work in different colour spaces, a particular RGB value in the model may represent different visual colours. Therefore, colour management concepts are required to develop a conversion between the colour spaces, and so allow a 3D colour reproduction system to produce chromatic dental models with colours visibly perceived to be accurate.

This study sought to develop a 3D colour reproduction system based on intraoral scanning and 3D printing. Conventional colour management methods were applied to an intra oral scanner and a 3D printer to establish their colour profiles. These profiles allowed colour data to be transferred from device-dependent colour spaces to a device-independent colour space and vice versa. An *in vitro* evaluation of tooth and gum shades then verified the feasibility and accuracy of this system in dentistry.

## Materials and Methods

### Protocol for 3D colour reproduction

**Figure 1:**
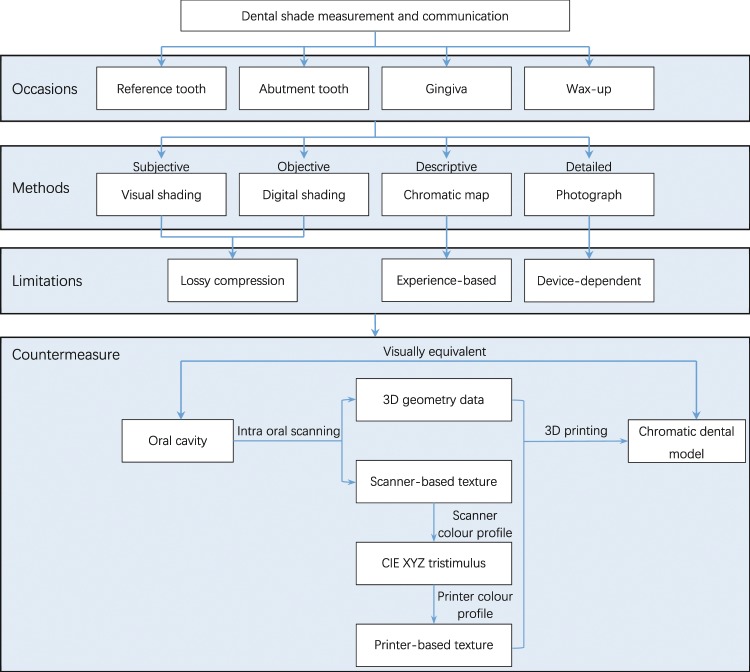
Scheme of the study.

An intra oral scanner (TRIOS 3, 3Shape A/S, Denmark) and a 3D printer (J750, Stratasys Ltd., Israel) were used to build the 3D colour reproduction system. The 3D digital model was captured using the scanner, and saved as a virtual reality modelling language (VRML) file composed of geometry data and texture images. The scanner-based RGB values of each pixel from the texture image were converted to device-independent CIE XYZ tristimulus values using the scanner colour profile, and then to printer-based RGB values using the printer colour profile. The original scanner-based texture images of the VRML model file were replaced with the converted printer-based texture images. Finally, the modified 3D digital model was printed using the 3D printer to produce a physical 3D colour model (Fig. 1).

### Development of colour profile for intra oral scanner

A crucial part of this research was the development of the colour profile for the intra oral scanner. This profile would link the RGB values of the scanner’s texture image to device-independent CIE XYZ tristimulus values.

**Figure 2:**
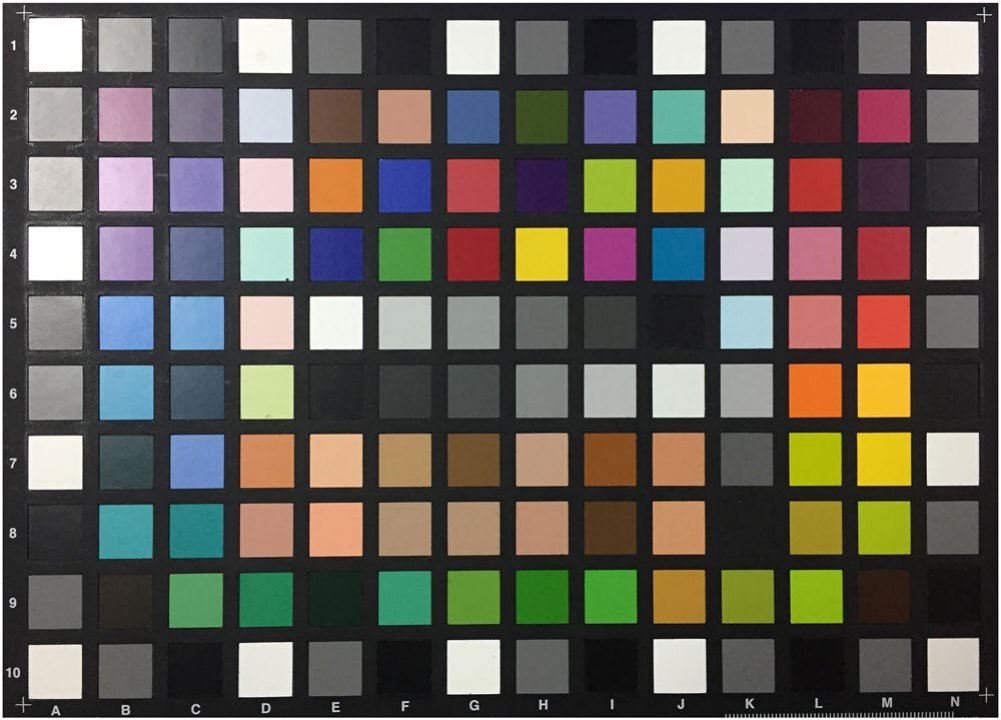
Conventional colour chart used for colour profile development.

A colour chart (ColorChecker Digital SG, X-Rite Inc., USA) (Fig. 2) provided colour patches. Excluding the outermost greyscale patches, 96 patches were selected from the chart for this process. The spectral reflectance of each patch was measured using a spectrophotometer (ColorEye 7000A, X-Rite Inc., USA). The CIE standard D65 illuminant [*I*(*λ*)] and 2° standard observer colour matching functions [$\bar{x}(\lambda )$, $\bar{y}(\lambda )$ and $\bar{z}(\lambda )$] were selected to calculate CIE XYZ tristimulus values based on the spectral data [*S*(*λ*)]:

$$\begin{array}{rcl}X & = & \frac{100}{N}{\int }_{380}^{780}\,S(\lambda )I(\lambda )\bar{x}(\lambda )d\lambda ,\\ Y & = & \frac{100}{N}{\int }_{380}^{780}\,S(\lambda )I(\lambda )\bar{y}(\lambda )d\lambda ,\\ Z & = & \frac{100}{N}{\int }_{380}^{780}\,S(\lambda )I(\lambda )\bar{z}(\lambda )d\lambda ,\end{array}$$

where

$$N={\int }_{380}^{780}\,I(\lambda )\bar{y}(\lambda )d\lambda .$$

The D65 illuminant and 2° standard observer were selected to simulate the practical requirements of dental shading which is generally bright neutral daylight and with a distance of about 1.5 m.

**Figure 3:**
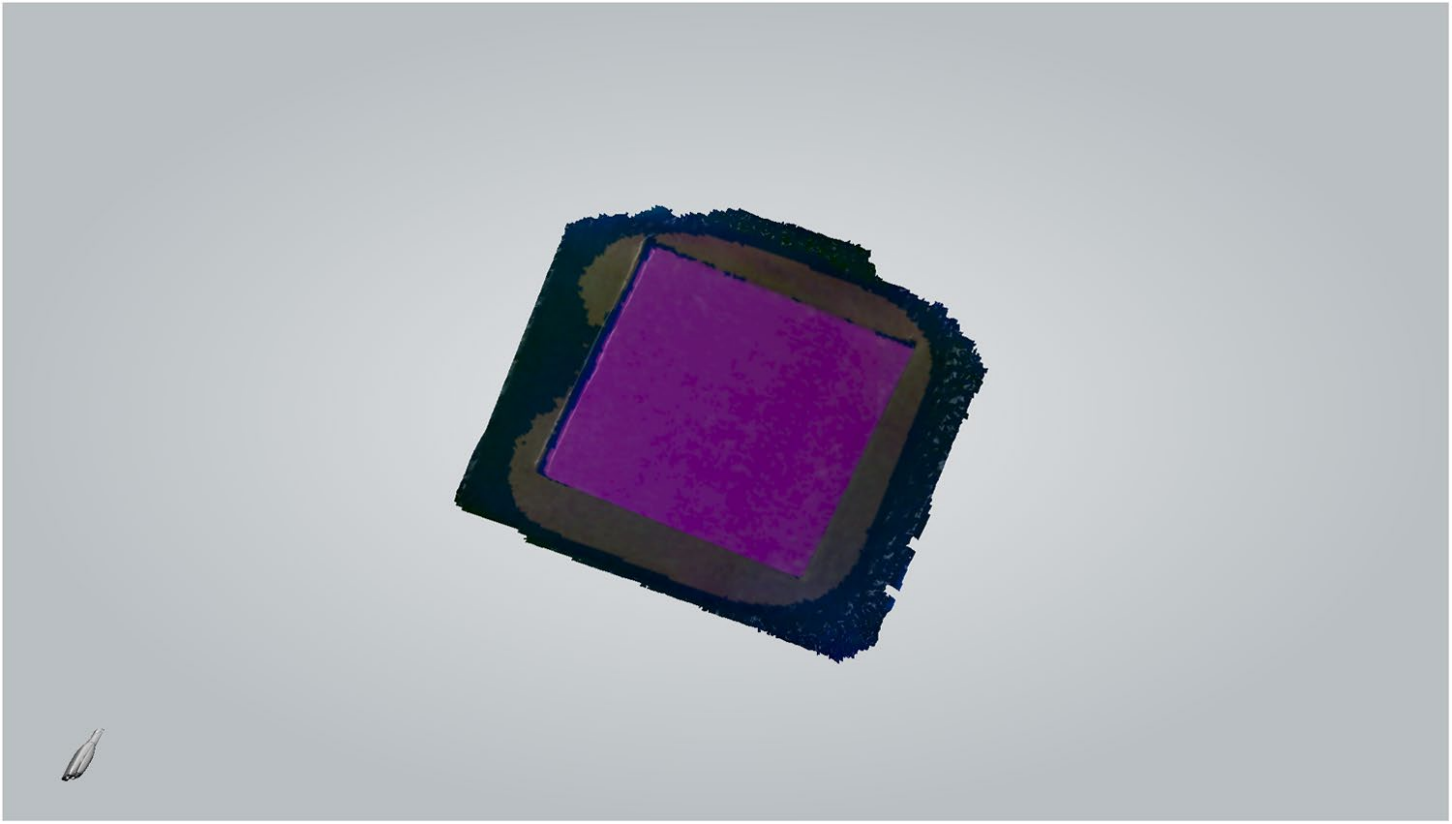
Colour patch digitized by the intra oral scanner.

**Figure 4:**
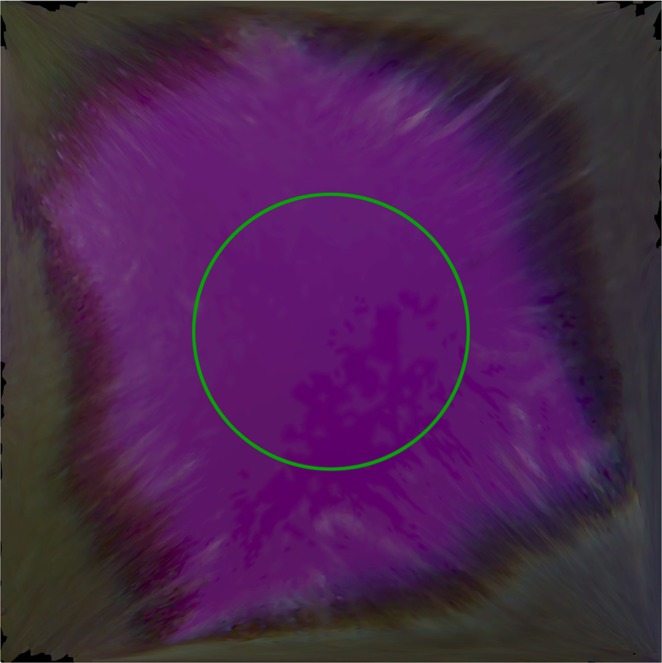
Texture image captured by the intra oral scanner. The green circle marks the area selected for RGB measurement.

Each patch was then digitized by the intra oral scanner calibrated according to the manual. The “quality” of the colour acquired by the scanner was monitored using the built-in “Shade” function. Any target area marked blue would undergo additional scans from different angles to get correct colour representation (Fig. 3). The digital models were saved as VRML files. The central circular area of each texture image (radius 425 pixels) containing 567,460 pixels (Fig. 4) was selected to calculate average RGB values using Photoshop CC 2014 (Adobe Systems Inc., USA). To normalize the data sets, CIE XYZ tristimulus values were divided by the white point of D65 illuminant (95.047, 100, and 108.883) and the RGB values were divided by 255.

The colour profile represents a mathematical model calculated to relate the original RGB values and the target CIE XYZ tristimulus values. Conventional polynomial regression based on least squares is chosen for its convenient implementation and accurate results^9–11^. In this case, the mathematical relationship can be represented as

$$T=OM,$$

where *T* denotes an *n* × 3 matrix composed of the normalized CIE XYZ tristimulus values of the target colour patch, *O* denotes an *n* × *p* matrix composed of the normalized RGB values and their polynomial terms of the texture image (where *n* is the number of colour patches and *p* is the number of terms in the polynomial), and *M* is the transfer matrix we seek. Therefore, the least-square solution of *M* is

$$M={({O}^{T}O)}^{-1}{O}^{T}T.$$

For simple linear regression, each row of *O* represents the normalized RGB values and a constant (usually 1) of a colour patch. The number of terms *p* therefore is 4, and

$$O=[\begin{array}{cccc}1 & {R}_{1} & {G}_{1} & {B}_{1}\\ \vdots  & \vdots  & \vdots  & \vdots \\ 1 & {R}_{n} & {G}_{n} & {B}_{n}\end{array}].$$

However, simple linear regression often cannot produce results with acceptable accuracy, thus quadratic and cubic polynomial regressions with more terms are considered. For the quadratic polynomial regression, *p* is 10, and

$$O=[\begin{array}{cccccccccc}1 & {{\rm{R}}}_{1} & {{\rm{G}}}_{1} & {B}_{1} & {R}_{1}^{2} & {G}_{1}^{2} & {B}_{1}^{2} & {{\rm{R}}}_{1}{{\rm{G}}}_{1} & {{\rm{R}}}_{1}{B}_{1} & {{\rm{G}}}_{1}{B}_{1}\\ \vdots  & \vdots  & \vdots  & \vdots  & \vdots  & \vdots  & \vdots  & \vdots  & \vdots  & \vdots \\ 1 & {{\rm{R}}}_{n} & {{\rm{G}}}_{n} & {B}_{n} & {{\rm{R}}}_{{\rm{n}}}^{2} & {{\rm{G}}}_{{\rm{n}}}^{2} & {{\rm{B}}}_{{\rm{n}}}^{2} & {{\rm{R}}}_{n}{{\rm{G}}}_{n} & {{\rm{R}}}_{n}{B}_{n} & {{\rm{G}}}_{n}{B}_{n}\end{array}].$$

For the cubic polynomial regression, *p* is 20, and

$$O=[\begin{array}{cccccccccccccccccccc}1 & {{\rm{R}}}_{1} & {{\rm{G}}}_{1} & {{\rm{B}}}_{1} & {{\rm{R}}}_{1}^{2} & {{\rm{G}}}_{1}^{2} & {{\rm{B}}}_{1}^{2} & {{\rm{R}}}_{1}{{\rm{G}}}_{1} & {{\rm{R}}}_{1}{{\rm{B}}}_{1} & {{\rm{G}}}_{1}{{\rm{B}}}_{1} & {{\rm{R}}}_{1}^{3} & {{\rm{G}}}_{1}^{3} & {{\rm{B}}}_{1}^{3} & {{\rm{R}}}_{1}^{2}{{\rm{G}}}_{1} & {{\rm{R}}}_{1}^{2}{{\rm{B}}}_{1} & {{\rm{G}}}_{1}^{2}{{\rm{R}}}_{1} & {{\rm{G}}}_{1}^{2}{{\rm{B}}}_{1} & {{\rm{B}}}_{1}^{2}{{\rm{R}}}_{1} & {{\rm{B}}}_{1}^{2}{{\rm{G}}}_{1} & {{\rm{R}}}_{1}{{\rm{G}}}_{1}{{\rm{B}}}_{1}\\ \vdots  & \vdots  & \vdots  & \vdots  & \vdots  & \vdots  & \vdots  & \vdots  & \vdots  & \vdots  & \vdots  & \vdots  & \vdots  & \vdots  & \vdots  & \vdots  & \vdots  & \vdots  & \vdots  & \vdots \\ 1 & {{\rm{R}}}_{n} & {{\rm{G}}}_{n} & {B}_{n} & {{\rm{R}}}_{{\rm{n}}}^{2} & {{\rm{G}}}_{{\rm{n}}}^{2} & {{\rm{B}}}_{{\rm{n}}}^{2} & {{\rm{R}}}_{n}{{\rm{G}}}_{n} & {{\rm{R}}}_{n}{B}_{n} & {{\rm{G}}}_{n}{B}_{n} & {{\rm{R}}}_{{\rm{n}}}^{3} & {{\rm{G}}}_{{\rm{n}}}^{3} & {{\rm{B}}}_{{\rm{n}}}^{3} & {{\rm{R}}}_{{\rm{n}}}^{2}{{\rm{G}}}_{{\rm{n}}} & {{\rm{R}}}_{{\rm{n}}}^{2}{{\rm{B}}}_{{\rm{n}}} & {{\rm{G}}}_{{\rm{n}}}^{2}{{\rm{R}}}_{{\rm{n}}} & {{\rm{G}}}_{{\rm{n}}}^{2}{{\rm{B}}}_{{\rm{n}}} & {{\rm{B}}}_{{\rm{n}}}^{2}{{\rm{R}}}_{{\rm{n}}} & {{\rm{B}}}_{{\rm{n}}}^{2}{{\rm{G}}}_{{\rm{n}}} & {{\rm{R}}}_{{\rm{n}}}{{\rm{G}}}_{{\rm{n}}}{{\rm{B}}}_{{\rm{n}}}\end{array}].$$

In theory, there is no limitation on the order and number of terms in the polynomial. However, considering the number of samples available, the cost of computing and the required accuracy, the polynomial was constrained. Previous works^9–13^ indicate that quadratic and cubic polynomials might be suitable here. Adjusted R-squares were adopted for both models to evaluate their accuracy. All the calculations were performed via programming using the C++/Qt5 and C++/Eigen3 libraries.

### Development of colour profile for 3D printer

Another crucial part of this research was the development of colour profile for the 3D printer. Different from the scanner colour profile, the printer profile would convert the device-independent CIE XYZ tristimulus values to printer-based RGB values.

**Figure 5:**
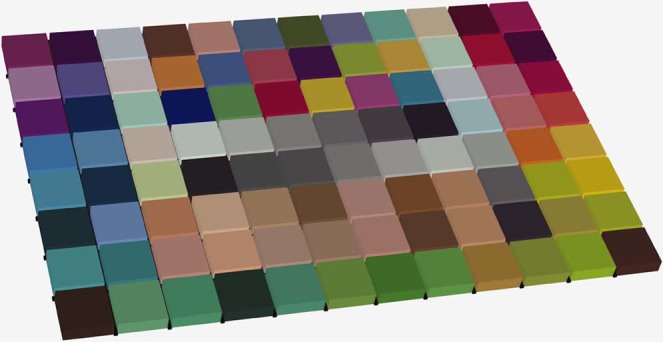
Reconstructed 3D colour chart.

**Figure 6:**
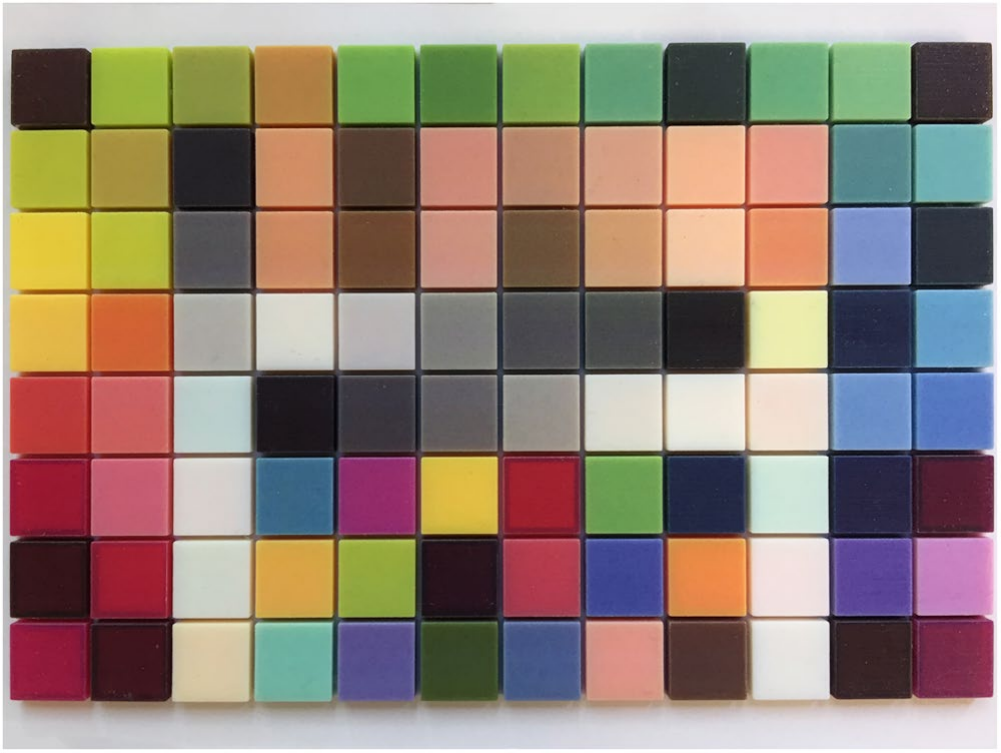
Directly printed 3D colour chart.

The digital models of the colour patches were cropped and reconstructed to blocks of 13 mm (length) * 13 mm (width) * 4 mm (height), and were aligned together to form a 3D colour chart (Fig. 5). The colour chart was printed by the 3D printer using its “High Quality” and matte surface finishing mode (Fig. 6). The spectral reflectance of each block of the 3D colour chart was also measured using the spectrophotometer and was calculated to CIE XYZ tristimulus values under the same condition. The relationship between these CIE XYZ tristimulus values of the printed blocks and their corresponding RGB values was also resolved using polynomial regression based on least squares, as described above, in which the target matrix *T* was composed of the normalized RGB values of the texture images and the original matrix *O* was composed of the normalized CIE XYZ tristimulus values and their polynomial terms for the printed blocks. Both quadratic and cubic polynomials were resolved. Adjusted R-squares were adopted for both models to evaluate their accuracy.

### Preliminary evaluation of the 3D colour reproduction system

As the CIE XYZ colour space is not particularly perceptually uniform, in order to evaluate the reproduction accuracy in terms of human visual perception, the CIE XYZ tristimulus values of the original colour chart and the printed colour chart are transformed to the CIE L*a*b* colour space according to the following formulas:

$$\begin{array}{rcl}{L}^{\ast } & = & 116f(\frac{Y}{{Y}_{n}})-16,\\ {a}^{\ast } & = & 500(f(\frac{X}{{X}_{n}})-f(\frac{Y}{{Y}_{n}})),\\ {b}^{\ast } & = & 200(f(\frac{Y}{{Y}_{n}})-f(\frac{Z}{{Z}_{n}})),\end{array}$$

where

$$\begin{array}{rcl}f(t) & = & \{\begin{array}{cc}\sqrt[3]{t}, & t > {\delta }^{3},\\ \frac{t}{3{\delta }^{2}}+\frac{4}{29}, & {\rm{otherwise}},\end{array}\\ \delta  & = & \frac{6}{29}.\end{array}$$

For D65 illuminant white point, *X*_*n*_ = 95.047, *Y*_*n*_ = 100 and *Z*_*n*_ = 108.883.

The difference of colours between the corresponding patches from the original 2D colour chart and the directly printed 3D colour charts can be calculated:

$$\Delta {E}_{ab}^{\ast }=\sqrt{\Delta {L}^{\ast 2}+\Delta {a}^{\ast 2}+\Delta {b}^{\ast 2}}.$$

where $\Delta {L}^{\ast }$, $\Delta {a}^{\ast }$ and $\Delta {b}^{\ast }$ respectively represent the differences of the two colours in the L*, a*, and b* parameters.

Given the compatibility of the profiles of the scanner and the printer, different profile combination might result in different colour reproduction performance. In order to select the most suitable combination of profiles, the texture images of the 3D colour chart were adjusted using four profile combinations: quadratic scanner profile - quadratic printer profile (QS-QP), quadratic scanner profile - cubic printer profile (QS-CP), cubic scanner profile - quadratic printer profile (CS-QP) and cubic scanner profile - cubic printer profile (CS-CP). The four adjusted 3D colour charts were printed using the configuration described above. The colour difference of each patch in the 3D colour charts was also measured and calculated. The combination with the lowest overall colour difference (CS-CP) was chosen for *in vitro* evaluation of tooth and gum shades.

### *In vitro* evaluation of tooth and gum shades of the 3D colour reproduction system

After different colour profiles were created and evaluated, the CS-CP profile combination was chosen to test on tooth and gum shades for the assessment of dental applications. The intraoral scanner digitized 18 resin blocks, including 16 body shades (A1 to D4) and two gum shades (GUM-L and GUM-D) (Ceramage, Shofu Inc., Japan). The digital models were reconstructed, and the textures were adjusted according to the workflow in Fig. 1. The reconstructed and adjusted models were printed by the 3D printer. The CIE L*a*b* values of the original resin blocks and the printed samples were calculated as described above. Calculation of the corresponding $\Delta {E}_{ab}^{\ast }$ evaluated the accuracy of this 3D colour reproduction system.

### Statistical analysis

To evaluate the difference of $\Delta {E}_{ab}^{\ast }$ for each profile combination, the non-parametric Friedman test was used following the Shapiro–Wilk test of normal distribution. The null hypothesis was that $\Delta {E}_{ab}^{\ast }$ for each profile combination presented no difference. The results of *in vitro* evaluation for tooth and gum shades were simply analysed using the mean and median considering the small sample size.

## Results

### Colour profiles for the intra oral scanner and the 3D printer

**Figure 7:**
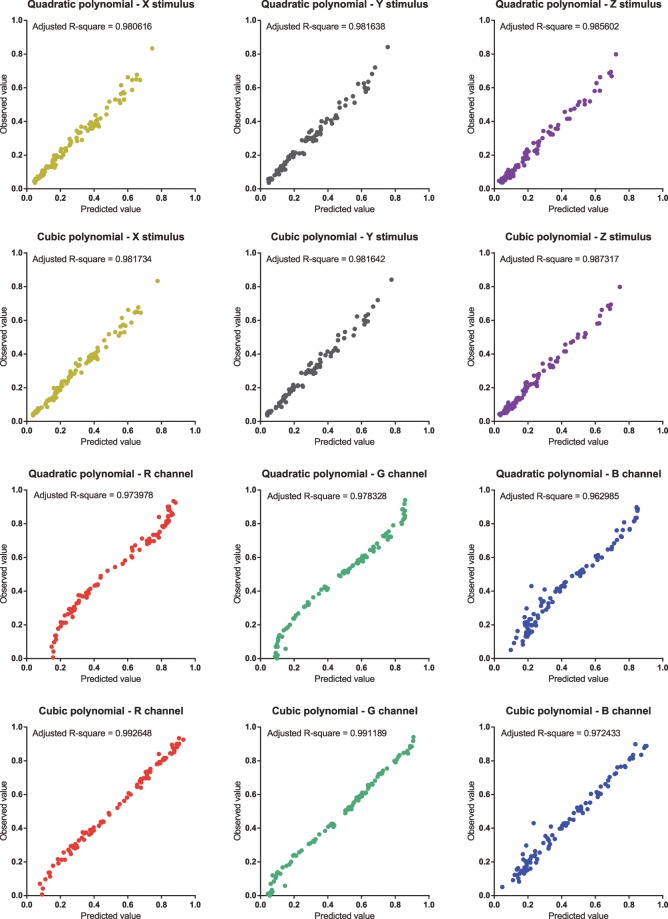
Scatter plots of predicted and observed values for colour profiles. Having more points closer to the diagonal indicates better fitting.

Two colour profiles for each device were developed, and their adjusted R-squares are given in Table 1. The means of the differences, $\Delta {E}_{ab}^{\ast }$, between the original and predicted values of the scanner colour profile are also provided. Increasing the order and number of terms achieved a better improvement for the 3D printer than for the intra oral scanner. The observed and predicted values for each patch using the colour profiles are plotted in Fig. 7. The adjusted R-squares and plots show both scanner colour profiles achieved good performance, whereas the cubic polynomial achieved better fitting for the printer colour profile than the quadratic polynomial.

**Table 1:** Adjusted R-squares of different channels of colour profiles.

	Intra oral scanner				3D printer		
	X-RGB	Y-RGB	Z-RGB	Mean ${\boldsymbol{\Delta }}{{\boldsymbol{E}}}_{{\boldsymbol{ab}}}^{\ast }$	R-XYZ	G-XYZ	B-XYZ
Quadratic polynomial	0.980616	0.981638	0.985602	4.3468	0.973978	0.978328	0.962985
Cubic polynomial	0.981734	0.981642	0.987317	3.7722	0.992648	0.991189	0.972433

### Difference of colours between the original 2D colour chart and the printed 3D colour charts

Table 2 lists the means, medians, standard deviations (SDs), and minima and maxima of $\Delta {E}_{ab}^{\ast }$ between the original 2D colour chart and the printed 3D colour charts.

**Table 2:** Means, SDs, and minima and maxima of ${\Delta E}_{{ab}}^{\ast }$ between the original and the printed colour charts.

	Mean	Median	SD	Minimum	Maximum
Directly printed	10.6567	8.2956^a,b,c^	7.5359	1.1381	34.6066
QS-QP	10.7402	9.2332^a^	6.3887	1.7190	35.0860
QS-CP	9.1396	7.1421^b^	6.2987	0.6333	33.7602
CS-QP	10.2818	8.5120^c^	6.4067	0.4851	36.4285
CS-CP	8.6611	6.9402^d^	6.3337	1.5040	32.6600

^*^SD = standard deviation.

**Different letters in superscript indicate statistically significant differences between groups (*P* < 0.005).

The Shapiro–Wilk test indicated that all five groups did not follow the normal distribution (*P* < 0.001); therefore, non-parametric Friedman testing was used for analysis. *Post hoc* comparisons were performed using the Wilcoxon signed ranks test with Bonferroni adjustment (*P* < 0.005). The results showed statistical differences among the five groups. Group CS-CP showed the smallest median and statistically significant difference with directly printed group, which indicated that this combination of colour profiles performed best.

### Difference of colours of reproduced tooth and gum shades

The CIE L*a*b* values and $\Delta {E}_{ab}^{\ast }$ of each pair of shade block are listed in Table 3. The values $\Delta {E}_{ab}^{\ast }$ ranged from 2.1850 to 11.2156 with a mean of 6.5378 and a median of 6.3129. The results matched the CS-CP profile described above. There was a tendency that darker tooth shades would result in larger errors. Based on the study of Johnston^14^, only A1 and B1 blocks achieved imperceptible colour difference ($\Delta {E}_{ab}^{\ast }$ < 3.70), while blocks A3.5, A4, B3, B4, C3, C4, D4 and GUM-L blocks has differences above the mismatch threshold ($\Delta {E}_{ab}^{\ast }$ > 6.80).

**Table 3:** CIE L*a*b* values of original and printed tooth and gum shade blocks.

	Original resin blocks			Printed blocks			ΔL*	Δa*	Δb*	${\boldsymbol{\Delta }}{{\boldsymbol{E}}}_{{\boldsymbol{ab}}}^{\ast }$
	L*	a*	b*	L*	a*	b*				
A1	74.6828	−0.5933	13.4117	72.8212	0.5421	13.2719	1.8616	−1.1354	0.1398	2.1850
A2	71.3588	0.4755	17.1232	67.4742	1.5797	16.4414	3.8846	−1.1042	0.6818	4.0956
A3	70.0063	1.5521	18.0021	67.1391	3.5635	21.7107	2.8672	−2.0114	−3.7086	5.1010
A3.5	65.1918	2.4270	18.5095	63.1816	−2.1094	23.5238	2.0102	4.5365	−5.0143	7.0543
A4	61.0780	3.0341	17.4426	59.6044	−2.8634	24.0564	1.4736	5.8976	−6.6138	8.9830
B1	74.9136	−1.6045	13.6834	73.3496	0.2086	13.2603	1.5640	−1.8131	0.4231	2.4316
B2	72.2844	−0.7184	15.4806	69.2184	2.9631	16.4467	3.0660	−3.6815	−0.9661	4.8874
B3	68.1883	1.5216	21.1903	66.1553	1.9898	28.6032	2.0330	−0.4682	−7.4129	7.7009
B4	67.7520	2.6461	23.1152	66.8389	4.3657	31.2590	0.9131	−1.7197	−8.1438	8.3733
C1	70.3434	0.0019	14.1987	65.5508	−1.1409	15.5456	4.7926	1.1427	−1.3469	5.1077
C2	67.7152	0.0926	17.7751	64.7726	−2.9067	22.5435	2.9426	2.9993	−4.7684	6.3555
C3	62.6822	1.4473	16.2507	61.3523	−3.4635	22.3806	1.3299	4.9109	−6.1299	7.9662
C4	60.5066	3.2225	20.0900	59.4054	−1.8050	30.0572	1.1012	5.0275	−9.9672	11.2176
D2	70.7429	0.1457	11.661	65.8538	−0.6209	11.6252	4.8891	0.7666	0.0359	4.9490
D3	69.4998	0.3323	15.6499	65.0438	−1.5804	17.9767	4.4560	1.9127	−2.3268	5.3785
D4	66.7409	1.1536	19.8001	62.7634	−3.8655	25.9416	3.9775	5.0191	−6.1415	8.8730
GUM-L	62.3765	22.0405	11.4103	60.9537	25.9975	21.3041	1.4228	−3.9570	−9.8938	10.7503
GUM-D	49.4025	24.3317	7.7882	48.7339	22.4012	13.7164	0.6686	1.9305	−5.9282	6.2704
Mean										6.5378
Median										6.3129

## Discussion

This study developed a method of building a 3D colour reproduction system and evaluated the accuracy of its colour reproduction. The resulting colour differences were unsatisfactory, possible owing to the cumulative errors from scanning, converting and printing. Related works have shown the intraoral scanner used here to achieve acceptable accuracy and precision in shade measurement^6–8^. This is confirmed by the scanner colour profile representing an acceptable mean of $\Delta {E}_{ab}^{\ast }$. However, the introduction of printing error made the system’s overall error unacceptable. The results resemble the outcome of traditional colour management using camera, scanner and printer. Digital cameras and desktop scanners based on additive colour models (e.g. RGB colour model) usually achieve good polynomial relationships between RGB and CIE standards^9,10,15^ with little prediction error. This is not the case for printers because of the properties of their colour model. Desktop printers usually use subtractive colour models (e.g. CMYK, Cyan, Magenta, Yellow and Key/Black) that absorb lights of certain wavelength from the ambient light, and reflect the others to the observers’ eyes. Subtractive colour models usually lead to a non-linear or non-polynomial relationship between the input RGB and output CIE standards^11,16^. The 3D printer employed here uses a similar CMYK colour model but with an additional white base material. Moreover, a CMYK colour model usually has a smaller gamut than RGB colour model and the CIE standard, which means that certain colours cannot be produced by the 3D printers^17^. Despite these shortcomings, the method employed here did decrease the colour difference of the reproduction result, and indicates the potential of further optimization.

**Figure 8:**
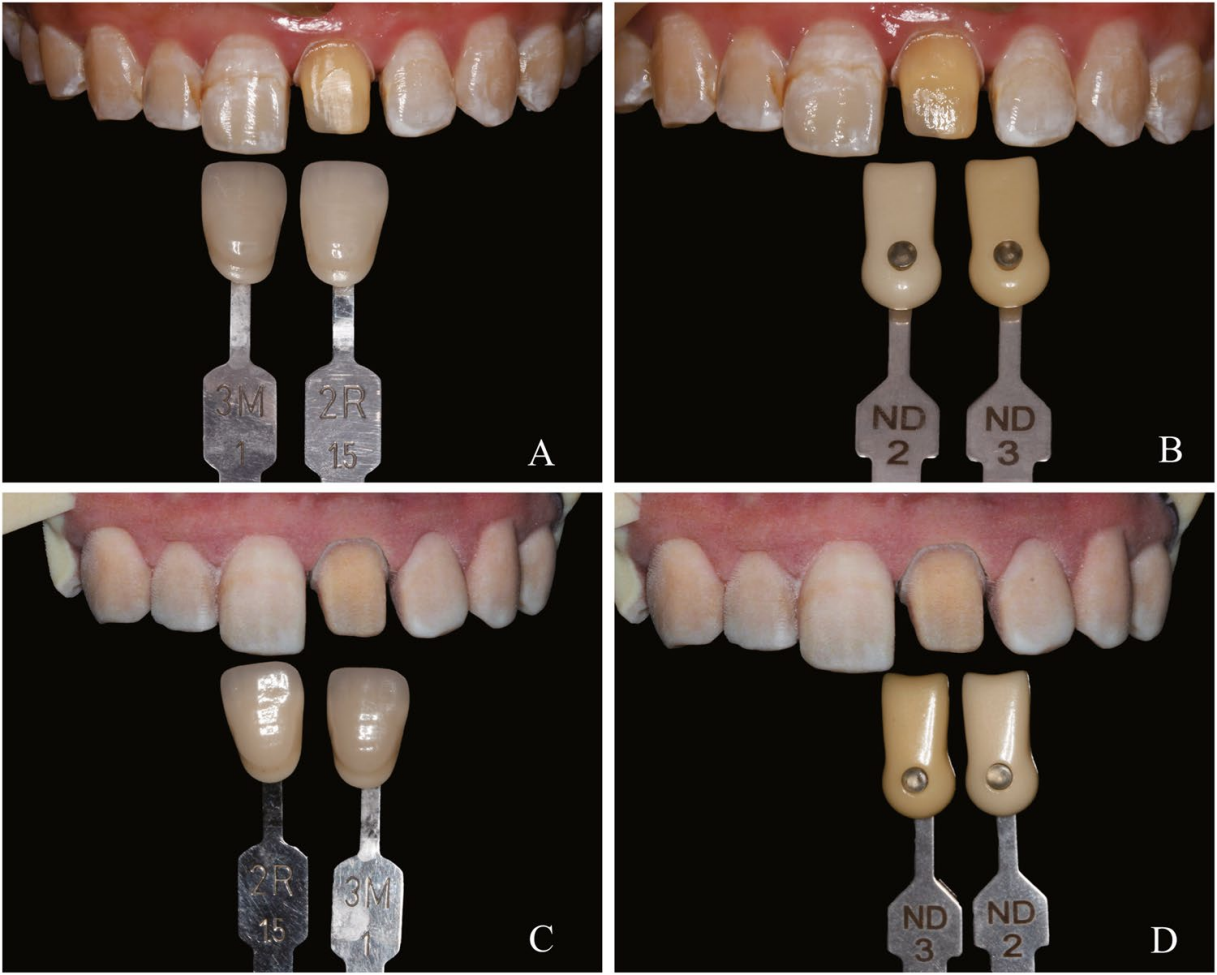
A preliminary clinical application of 3D printed chromatic model. A and B, intraoral view. C and D, printed model. The shade tabs were used to record the actual tooth shade and the model was used to demonstrate the feature of texture. Despite perceptible colour difference, the dental technician could visually locate the brown band and white spot lesion on the enamel surfaces.

Although this 3D colour reproduction system cannot transfer with sufficient accuracy shade information from a patient’s oral cavity to dental models for prostheses fabrication, some situations might have less stringent accuracy requirement. The 3D colour printed models can bring more detailed shade information of the natural abutment than a natural die shade guide and materials. For cases requiring the reproduction of complex textures of adjacent teeth or even gingiva and mucosa, the chromatic dental models can help dental technicians to locate these features on adjacent teeth and restorations in a visualized way (Fig. 8). The chromatic dental models may also be used as advanced wax-ups to enhance communication with patients.

To enhance the performance of the 3D colour reproduction system, besides improving the accuracy of the hardware, developing new colour profiles with higher precision would also be useful. Increasing the amount of training samples for the polynomial regression, particularly by adding more dental shades, may be the most straightforward way. Other methods such as using neural networks and lookup tables similar to traditional colour management workflows can also be introduced.

## Conclusions

This study investigated a colour management method based on polynomial regression to develop a novel 3D colour reproduction system based on intraoral scanning and 3D printing. While the system’s use of polynomial regression can improve colour reproduction, it currently remains unable to achieve satisfactory results in terms of colour difference. Further improvements are needed in the form of advanced colour management methods and suitable hardware. The potential applications of the chromatic models also need further exploration.

## Data Availability

The datasets generated during and/or analysed during the current study are available from the corresponding author on reasonable request.
